# Supplementary material for: The causal relationship between the human gut microbiota and pyogenic arthritis: a Mendelian randomization study
Source: Front Cell Infect Microbiol. 2024 Nov 26;14:1452480. doi: 10.3389/fcimb.2024.1452480 (PMC11629706; doi:10.3389/fcimb.2024.1452480)
Supplement: Supplementary file 1 [file DataSheet1.zip › mendelian test/ebi-a-GCST90027603.csv_forest.pdf]

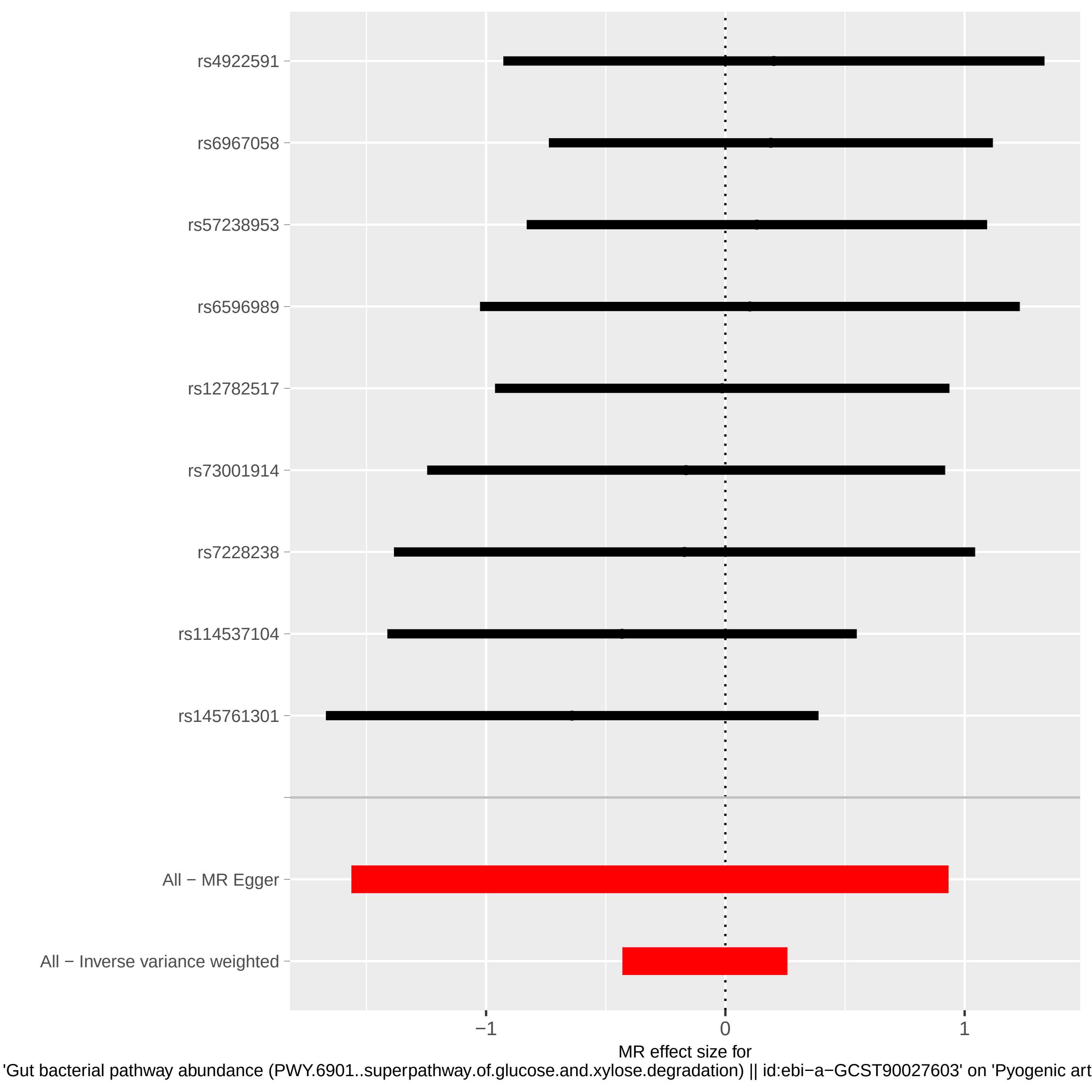

rs4922591
rs6967058
rs57238953
rs6596989
rs12782517
rs73001914
rs7228238
rs114537104
rs145761301
All − MR Egger
All − Inverse variance weighted
−1
0
1
MR effect size for
'Gut bacterial pathway abundance (PWY.6901..superpathway.of.glucose.and.xylose.degradation) || id:ebi−a−GCST90027603' on 'Pyogenic art
